# Novel deep learning-based noise reduction technique for prostate magnetic resonance imaging

**DOI:** 10.1007/s00261-021-02964-6

**Published:** 2021-02-12

**Authors:** Xinzeng Wang, Jingfei Ma, Priya Bhosale, Juan J. Ibarra Rovira, Aliya Qayyum, Jia Sun, Ersin Bayram, Janio Szklaruk

**Affiliations:** 1 MR Clinical Solutions and Research Collaborations, GE Healthcare, Houston, TX USA; 2 Department of Imaging Physics, The University of Texas MD Anderson Cancer Center, Houston, TX, USA, 1515 Holcombe Blvd., Houston, TX USA; 3 Department of Abdominal Imaging, The University of Texas MD Anderson Cancer Center, Houston, TX, USA, 1515 Holcombe Blvd., Houston, TX USA; 4 Department of Biostatistics, The University of Texas MD Anderson Cancer Center, Houston, TX, USA, 1515 Holcombe Blvd., Houston, TX USA

**Keywords:** Prostate, Deep learning, Magnetic resonance, Cancer, Endorectal coil

## Abstract

**Introduction:**

Magnetic resonance imaging (MRI) has played an increasingly major role in the evaluation of patients with prostate cancer, although prostate MRI presents several technical challenges. Newer techniques, such as deep learning (DL), have been applied to medical imaging, leading to improvements in image quality. Our goal is to evaluate the performance of a new deep learning-based reconstruction method, “DLR” in improving image quality and mitigating artifacts, which is now commercially available as AIR^TM^ Recon DL (GE Healthcare, Waukesha, WI). We hypothesize that applying DLR to the T2WI images of the prostate provides improved image quality and reduced artifacts.

**Methods:**

This study included 31 patients with a history of prostate cancer that had a multiparametric MRI of the prostate with an endorectal coil (ERC) at 1.5 T or 3.0 T. Four series of T2-weighted images were generated in total: one set with the ERC signal turned on (ERC) and another set with the ERC signal turned off (Non-ERC). Each of these sets then reconstructed using two different reconstruction methods: conventional reconstruction (Conv) and DL Recon (DLR): ERC_DLR_, ERC_Conv_, Non-ERC_DLR_, and Non-ERC_Conv_. Three radiologists independently reviewed and scored the four sets of images for (i) image quality, (ii) artifacts, and (iii) visualization of anatomical landmarks and tumor.

**Results:**

The Non-ERC_DLR_ scored as the best series for (i) overall image quality (*p* < 0.001), (ii) reduced artifacts (*p* < 0.001), and (iii) visualization of anatomical landmarks and tumor.

**Conclusion:**

Prostate imaging without the use of an endorectal coil could benefit from deep learning reconstruction as demonstrated with T2-weighted imaging MRI evaluations of the prostate.

## Introduction

Prostate cancer affects one in six men during his lifetime and is the second leading cause of cancer death in the United States. Magnetic resonance imaging (MRI) has played an increasingly important role in the evaluation of patients with suspected or confirmed prostate cancer, especially in staging the disease [1, 2], such as in the detection of extraprostatic extension. More recently, MRI has been increasingly used in the detection of primary tumors in all patients, including “biopsy naïve” patients. Technical guidelines such as the Prostate Imaging Reporting and Data System (PI-RADS) have been instrumental in making these changes and in improving the interpretation of the MRIs of the prostate by establishing minimum standards for high-quality images and a protocol for optimal image interpretation.

The standard prostate MRI protocol consists of multiple sequences, including the T2-weighted (T2W) images, diffusion-weighted MRI (DWI), and dynamic contrast-enhanced (DCE) series with Gd-injection [3]. T2-weighted imaging (T2WI) is commonly performed in three orthogonal planes. These techniques are used to evaluate the primary malignancy and to stage the extent of disease.

Prostate MRI presents several technical challenges and controversies. Some commonly encountered challenges include a low signal-to-noise ratio (SNR, especially with scanners of a field strength of < 3 T) and the presence of artifacts that could compromise the image interpretation, such as the strong pulsation artifacts from the rectum. The use of an endorectal coil (ERC) for prostate MRI is controversial [4]. An ERC provides a definite advantage over surface array coils with a better SNR, which is essential for the high spatial resolution that is needed for accurate visualization of the prostate and its surrounding anatomy and for the detection of prostate cancer. However, ERC often has exacerbated motion artifacts due to its proximity to regions of interest (ROIs). The use of an ERC is also cumbersome, costly, and uncomfortable or even intolerable to some patients. From the patient perspective, discomfort can lead to noncompliance and to unwillingness to undergo future MRI studies and to the patient potentially missing an optimal window of intervention for managing the disease.

Deep learning (DL) and machine learning (ML) have recently experienced explosive growth in applications, including in many areas of medical research [5, 6]. For prostate MRI, ML and DL have been used successfully for prostate segmentation, cancer detection, evaluation of local aggressiveness, staging, pretreatment assessment, and biochemical recurrence [7]. DL techniques have also been reported for improved image quality and noise reduction [8, 9].

In this work, we applied a novel DL-based MRI reconstruction method (hereafter referred to as “DLR”) to clinical prostate T2W fast spin-echo prostate imaging [10].

Our goal was to evaluate the performance of this new reconstruction method in improving image quality and mitigating artifacts. We hypothesize that DLR-generated images can provide better image quality and fewer artifacts than do the images obtained by conventional image reconstruction algorithms (hereafter referred to as “Conv”). Our secondary goal was to evaluate DLR-generated image quality with and without the signal from the ERC. We hypothesize that DLR images can provide adequate image quality with fewer artifacts when the ERC signal is removed.

## Patients and methods

### Patient population

The patient population was selected from all patients that had complete Endorectal MR-exam of the prostate during a six-month period (3/2019–9/2019) on either 1.5 T or 3.0 T (GE Healthcare, Waukesha, WI). Patients with no history of biopsy-proven prostate cancer, prior prostatectomy, or therapy for prostate cancer were excluded from the study. This study was IRB approved and informed consent was waived due to the retrospective nature of the study.

### Deep learning reconstruction

As detailed in [10], the DLR technique consists of a deep convolutional network trained with a supervised learning approach using pairs of images representing near-perfect and conventional MRI images for noise removal and high in-plane resolution. The database of training images spans a broad range of image content, enabling generalizability of the network across all anatomies. The database consists of more than 10,000 images, and image augmentations (rotations, flips, etc.) were applied to create 4 million unique image/augmentation combinations for added robustness. The loss between the predicted and the near-perfect images was minimized using the ADAM optimizer. The network offered a tunable noise reduction factor to accommodate user preference. The DLR was based on a residual encoder. Instead of directly generating high SNR images, the residual encoder offered flexibility on noise reduction. This can help in avoiding aggressive denoising by adding back a little controlled level of noise, making it appear more natural to human eyes. The DLR network was embedded into the conventional reconstruction pathway such that two sets of image series could be generated from a single set of raw MRI data. Data for training were not acquired from the scanners used.

### MRI acquisition

All axial T2W fast spin-echo images were acquired with an external pelvic phased array coil in combination with an ERC. The typical scan parameters were *T*_E_/*T*_R_ = 133–150 ms/4000–8500 ms; *E*_TL_ = 24; 3/0 mm; 256 × 192; *N*_ex_ = 2 at either 1.5 T or 3.0 T. On two series, the signal from the ERC was turned off, without physically removing the coil from the patients. These images were labeled as Non-ERC. In two series, the DL reconstructions using the above-described model [10] were applied and these two series were labeled DLR. Thus, a total of four set of images were generated for each patient: (i) T2W with ERC with conventional image reconstruction (ERC_Conv_), (ii) Non-ERC T2W with conventional image reconstruction (Non-ERC_Conv_), (iii) T2W with ERC with DLR (ERC_DLR_), and (iv) Non-ERC T2W with DLR (Non-ERC_DLR_).

### Evaluation of images

Three radiologists, each with more than 15 years of experience in abdominal imaging, independently reviewed the set of four series. Images were displayed using a GE AW workstation (GE Healthcare, Waukesha, WI) by one radiologist while the others interpreted and scored the images. The radiologists did not review more than eight studies in one session to minimize the effect of fatigue. They were blinded to image acquisition details and to the location of biopsy-proven prostate cancer. In addition, the series were scrambled on the AW workstation to reduce bias: for example, Series 1 for patient “1” may correspond to ERC_Conv_, and Series 1 for patient “2” may correspond to Non-ERC_Conv_.

For each series, each reader separately scored the overall image artifacts on a 1-to-5 scale (1 = no artifacts to 5 = severe artifacts/nondiagnostic). This was scored based on each radiologist’s clinical experience on interpretation of the prostate MRIs. In addition, the readers also separately scored the overall image quality on a 1-to-3 scale (1 = excellent, 2 = adequate, 3 = poor) for each sequence. This was scored based on each radiologist’s clinical experience on interpretation of prostate MRIs. The individual assessment provided independent evaluation of image quality and artifacts of each series.

Comparing the four series side-to-side, each reader selected one or possibly two (if tied) of the best series for visualization of the prostate capsule (C), neurovascular bundle (NVB), anterior rectal wall (ARW), seminal vesicles (SV), ejaculatory duct (ED), urogenital diaphragm (UD), and tumor (T). A side-to-side comparison provided direct comparison between the four series. The reader also selected one or possibly two (if tied) worst series for visualization of C, NVB, ARW, SV, ED, UD, and T. The landmarks selected were those most commonly evaluated during the review of an MR prostate examination.

Each radiologist selected the tumor location independently. The radiologists were not aware of biopsy results. The radiologists selected a location that they suspect to represent tumor. The radiologists compared the visualization of the suspected tumor between the different series. The suspected tumor may not be same for all radiologists and may not correspond to the biopsy-proven location. The radiologists did not have access to the entire examination or to the electronic health records when performing the evaluations.

### Qualitative analysis

One radiologist placed a round ROI, at least 1 cm, on the peripheral zone (PZ), transitional zone (TZ), and obturator internus muscle (M) on each of the four series. The signal intensity from the ROI was considered as the signal for the corresponding anatomical location. The signal ratios for the ERC vs. Non-ERC series were calculated. The signal ratios of Conventional versus DLR for each anatomical location were also calculated.

### Interobserver variability

Interobserver variability was calculated for the scoring of overall image quality (excellent vs. adequate vs. poor) and for the artifacts (nondiagnostic, barely diagnostic, moderate, minimum, and no artifacts).

### Statistical analysis

Reader ratings were summarized by using frequencies and percentages. The generalized estimating equation method was used to assess the effect of methods on Likert scales, adjusted for the reader. The generalized estimating equation method takes into account the correlations among measurements from the same patient. *p*-values of pairwise comparisons were adjusted by using the Tukey–Kramer method to control the overall type I error rate. Interobserver agreement was assessed by using Krippendorff *α*, a generalization of the *κ* statistic. The *p*-value was considered statistically significant at less than 0.05.

## Results

### Patient population

Thirty-one patients were enrolled in this study. The average age was 67.3 years (range 49–82 years). The average and median prostate-specific antigen (PSA) values were 6.1 and 6.45 ng/mL, respectively (PSA range 1.5–25.7 ng/mL). The average and median Gleason scores were 7.8 and 7.5 (Gleason range 6–10). Fourteen patients were scanned at 1.5 T and 17 patients at 3.0 T.

### Denoising

In our study, a sample pilot project consisting of 5 patients was first conducted to compare images processed with various noise reduction levels (25%, 50%, 75%, and 100%). These images were evaluated by the 4 radiologists. Given a reasonable SNR in the original T2W images, all the radiologists preferred a 75% denoising level, which removed most of the noise without making the images appear overly synthetic: a balance between acceptable image quality and denoising.

### Artifacts

Table 1 shows the scores for image artifacts for the separate series: ERC_DLR_, ERC_Conv_, Non-ERC_DLR_, and Non-ERC_Conv_. Very few series were scored as barely diagnostic or nondiagnostic. None of the Non-ERC series were scored as barely diagnostic or nondiagnostic. When comparing 1.5 T and 3.0 T series, the 1.5 T Non-ERC series had a higher percentage of poor image quality. This was expected due to the lower signal-to-noise ratio without an ERC and lower field scanner. Using a point system of 1–5 [Non-diagnostic (ND = 1), Barely diagnostic (BD = 2), Moderate artifacts (MA = 3), Minimum artifacts (MA = 4), and No artifacts (NA = 5)]. The overall score for each series was calculated (Table 2, when a series was scored no artifacts, it received 5 points).

**Table 1:** Summary scoring of image artifacts for each series: Non-diagnostic (ND), Barely diagnostic (BD), Moderate artifacts (M), Minimum artifacts (MA), and No artifacts (NA)

	ND	BD	M	MA	NA	Magnetic field (T)
Non-ERC_Conv_	0	0	2	18	19	1.5
Non-ERC_Conv_	0	0	3	37	14	3
Non-ERC_Conv_	0	0	5	55	33	Combined
Non-ERC_DLR_	0	0	0	17	22	1.5
Non-ERC_DLR_	0	0	7	32	15	3
Non-ERC_DLR_	0	0	7	49	37	Combined
ERC_DLR_	0	4	19	17	0	1.5
ERC_DLR_	1	8	29	14	1	3
ERC_DLR_	1	12	48	31	1	Combined
ERC_Conv_	0	2	22	13	2	1.5
ERC_Conv_	2	7	29	16	0	3
ERC_Conv_	2	9	51	29	2	Combined

The columns correspond to the number of times each score was assigned to each series. The table list the results for the 1.5 T, 3.0 T, and the combined dataset. For example, Non-ERC_DLR_ score no artifacts, 37 times for the combined dataset. Non-ERC_Conv_ and Non-ERC_DLR_ were statistically superior to ERC_Conv_ and ERC_DLR_ (*p* < 0.001). No statistically significant differences were seen between ERC_Conv_ and ERC_DLR_ (*p* = 0.999)

**Table 2:** Summary scoring of image artifacts for each series, using the point scale 1-to-5 scale: Non-diagnostic (ND = 1), Barely diagnostic (BD = 2), Moderate artifacts (M = 3), Minimum artifacts (MA = 4), and No artifacts (NA = 5)

	Points	Magnetic field (T)
Non-ERC_Conv_	173	1.5
Non-ERC_Conv_	227	3.0
Non-ERC_Conv_	400	Combined
Non-ERC_DLR_	178	1.5
Non-ERC_DLR_	224	3.0
Non-ERC_DLR_	402	Combined
ERC_DLR_	133	1.5
ERC_DLR_	165	3.0
ERC_DLR_	298	Combined
ERC_Conv_	132	1.5
ERC_Conv_	167	3.0
ERC_Conv_	299	Combined

The results are shown for the 1.5 T, 3.0 T, and combined dataset. The overall point score for each series is shown below. The higher the score, the less artifacts affecting image quality

### Image quality

**Fig. 1:**
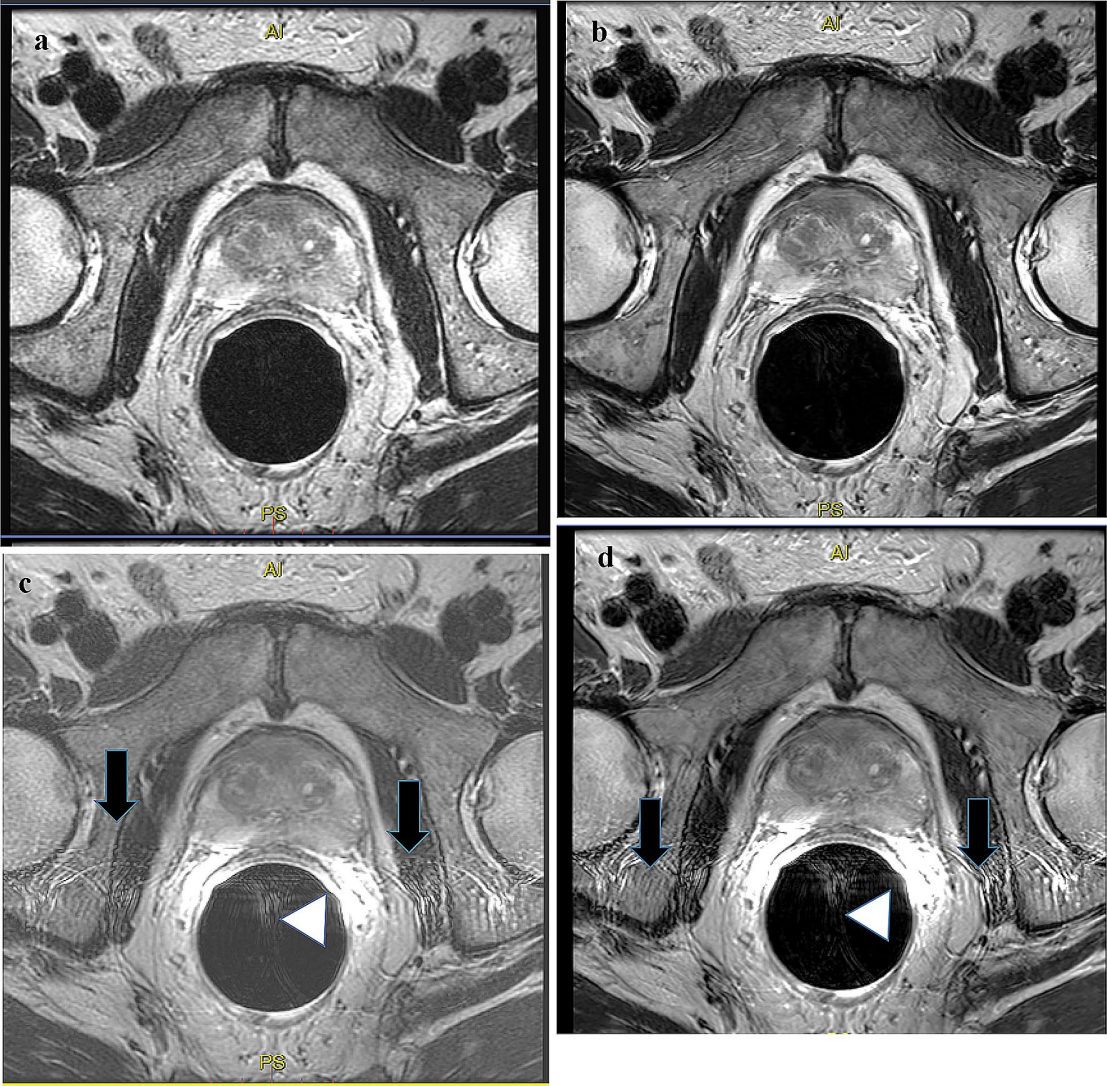
Axial T2-weighted images of a 61-year-old patient with a history of prostate cancer. **a** Images obtained with the ERC signal turned off and without DLR (Non-ERC_Conv_). **b** Images generated with the ERC signal turned off and with DLR (Non-ERC_DLR_). **c** Images obtained with the ERC signal turned on and without DLR (ERC_Conv_). **d** Images obtained with the ERC signal turned on and with DLR (ERC_DLR_). On **c** and **d**, note the pulsation artifacts from the coil (black arrows) and the near field artifacts (arrowhead) that are not seen on **a** or **b**

Figure 1 shows an example of the four series generated for each patient. Table 3 shows the score for overall image quality. The Non-ERC_DLR_ series received an excellent score 56.7% of the time. This was significantly better than the other series (*p* < 0.001). An ROI was placed in the PZ, TZ, and M (muscle). The signal ratios of the ERC images were 3.4 to 4.8 times higher than the signal ratios of the Non-ERC images. The signal ratios of the DLR and conventional were similar (1.0 and 1.4 times).

**Table 3:** The qualitative results for the best overall image quality

Field	1.5 T	1.5 T	1.5 T	3 T	3 T	3 T	Combined	Combined	Combined
Score	Poor	Adequate	Excellent	Poor	Adequate	Excellent	Poor	Adequate	Excellent
Non-ERC_Conv_	8	26	6	7	35	11	15	61	17
Non-ERC_DLR_	1	7	33	0	17	35	1	24	68
ERC_DLR_	0	29	10	5	38	11	5	67	21
ERC_Conv_	1	34	4	6	38	10	7	72	14

The number of series the radiologists selected the image quality as either excellent, adequate, or poor. The results are presented for 1.5 T, 3.0 T, and combined data for both magnetic fields. Non-ERC_DLR_ was scored as “excellent” in 56.7% of all the” excellent” series (68/120). These scores were better than those in the other three series, and differences were statistically significant (*p* < 0.001)

### Anatomical landmarks and tumor

Tables 4, 5, and 6 show the results for the qualitative analysis of the best series for the visualization of the ARW, UD, ED, C, NVB, and tumor. When a single series was selected, “Non-ERC_DLR_” was chosen more frequently as the “best series” for each category (55/72, 62/82, 63/78, 69/79, 65/80, and 67/80, respectively). For example, the ERC_DLR_ received a score of best series for visualization of the ARW only once. In contrast, the Non-ERC_DLR_ received the score as best series for ARW 55 times.

**Table 4:** Frequency of selection for each series as the “the best series” when only *one series* was selected for evaluation of the anterior rectal wall (ARW), urogenital diaphragm (UD), ejaculatory duct (ED), capsule and zonal anatomy (C), neurovascular bundle (NVB), and tumor (T)

	ERC_DLR_	ERC_Conv_	Non-ERC_DLR_	Non-ERC_Conv_
ARW	1	4	55	12
UD	5	7	62	8
ED	5	3	63	7
C	1	2	69	7
NVB	0	2	65	13
T	3	5	67	5

The Non-ERC_DLR_ was the commonly selected series for all categories

*ERC*_*Conv*_ Non-DLR processed images with ERC signal, *Non-ERC*_*Conv*_ Non-DLR processed images without ERC signal, *Non-ERC*_*DLR*_ DLR processed images without ERC signal, *ERC*_*DLR*_ DLR processed images with ERC signal

**Table 5:** Frequency of selection for each series as the “the best series” when tied with another series for the evaluation of anterior rectal wall (ARW), urogenital diaphragm (UD), ejaculatory duct (ED), capsule and zonal anatomy (C), neurovascular bundle (NVB), and tumor (T)

	ERC_DLR_	Non-ERC_DLR_	ERC_Conv_	Non-ERC_Conv_
ARW	3	20	2	17
UD	5	6	4	3
ED	4	12	3	7
C	2	13	4	9
NVB	1	12	0	11
T	8	11	3	5

The Non-ERC_DLR_ was the commonly selected series for all categories

*ERC*_*Conv*_ conventional reconstruction images with ERC signal, *Non-ERC*_*Conv*_ conventional reconstruction images without ERC signal, *Non-ERC*_*DLR*_ DLR processed images without ERC signal, *ERC*_*DLR*_ DLR processed images with ERC signal

**Table 6:** Frequency of selection for each series as the “the best series” when single or tied with another series for the evaluation of anterior rectal wall (ARW), urogenital diaphragm (UD), ejaculatory duct (ED), capsule and zonal anatomy (C), neurovascular bundle (NVB), and tumor (T)

	ERC_DLR_	ERC_Conv_	Non-ERC_DLR_	Non-ERC_Conv_
ARW	4	6	75	29
UD	10	11	68	11
ED	9	6	75	14
C	3	6	82	16
NVB	1	2	77	24
T	11	8	78	10

The Non-ERC_DLR_ was the commonly selected series for all categories

*ERC*_*Conv*_ conventional reconstruction images with ERC signal, *Non-ERC*_*Conv*_ conventional reconstruction images without ERC signal, *Non-ERC*_*DLR*_ DLR processed images without ERC signal, *ERC*_*DLR*_ DLR processed images with ERC signal

When two series were tied for the best series, “Non-ERC_DLR_” was also selected more frequently as “the best series” for each category: ARW, UD, ED, C, NVB, and T (20/42, 6/18, 12/26, 13/28, 12/24, and 11/27, respectively). For example, “Non-ERC_DLR_” received the score as best series for ARW 20 times when tied.

Tables 7, 8 and 9 show results for the qualitative analysis of the worst series for visualization of the ARW, UD, ED, C, NVB, and T. When a single series was selected, “Non-ERC_DLR_” was chosen least frequently as the “worst series” for each category (0/72, 4/84, 1/81, 1/72, 1/74, and 1/85, respectively).

**Table 7:** Frequency of selection for each series as the “the worse series” when only one series was selected for the evaluation of anterior rectal wall (ARW), urogenital diaphragm (UD), ejaculatory duct (ED), capsule and zonal anatomy (C), neurovascular bundle (NVB), and tumor (T)

	ERC_DLR_	ERC_Conv_	Non-ERC_DLR_	Non-ERC_Conv_
ARW	42	29	0	4
UD	12	21	4	47
ED	27	29	1	24
C	27	21	1	23
NVB	35	32	1	6
T	29	30	1	25

The Non-ERC_DLR_ was the least commonly selected series for all categories

*ERC*_*Conv*_ conventional reconstruction images with ERC signal, *Non-ERC*_*Conv*_ conventional reconstruction images without ERC signal, *Non-ERC*_*DLR*_ DLR processed images without ERC signal, *ERC*_*DLR*_ DLR processed images with ERC signal

**Table 8:** Frequency of selection for each series as the “the worse series” when tied with another series for the evaluation of anterior rectal wall (ARW), urogenital diaphragm (UD), ejaculatory duct (ED), capsule and zonal anatomy (C), neurovascular bundle (NVB), and tumor (T)

	ERC_DLR_	ERC_Conv_	Non-ERC_DLR_	Non-ERC_Conv_
ARW	56	43	1	5
UD	15	24	4	48
ED	31	36	1	27
C	43	38	1	25
NVB	50	48	1	7
Tumor	33	33	1	27

The Non-ERC_DLR_ was the least commonly selected series for all categories

*ERC*_*Conv*_ conventional reconstruction images with ERC signal, *Non-ERC*_*Conv*_ conventional reconstruction images without ERC signal, *Non-ERC*_*DLR*_ DLR processed images without ERC signal, *ERC*_*DLR*_ DLR processed images with ERC signal

**Table 9:** Frequency of selection for each series as the “the worse series” when single or tied with another series for the evaluation of anterior rectal wall (ARW), urogenital diaphragm (UD), ejaculatory duct (ED), capsule and zonal anatomy (C), neurovascular bundle (NVB), and tumor (T)

	ERC_DLR_	ERC_Conv_	Non-ERC_DLR_	Non-ERC_Conv_
ARW	98	72	1	9
UD	27	45	8	95
ED	58	65	2	51
C	70	59	2	48
NVB	85	80	2	13
Tumor	33	33	1	27

The Non-ERC_DLR_ was the least commonly selected series for all categories

*ERC*_*Conv*_ conventional reconstruction images with ERC signal, *Non-ERC*_*Conv*_ conventional reconstruction images without ERC signal, *Non-ERC*_*DLR*_ DLR processed images without ERC signal, *ERC*_*DLR*_ DLR processed images with ERC signal

When two series were tied for the worst series, “Non-ERC_DLR_” was also selected less frequently as “worst series” for categories ARW, UD, ED, C, NVB, and T (1/105, 4/91, 1/95, 1/107, 1/106, and 1/94, respectively). When combining the single and tied series as “worst series,” “Non-ERC_DLR_” was selected as the “the worst series” less frequently for each category (1/180, 8/175, 2/176, 2/179, 2/180, and 1/94). In contrast, “ERC_DLR_” was selected the worst of visualization of the ARW (98/180), C (70/179), and NVB (85/180). The “ERC_Conv_” and “ERC_DLR_” tied as the worst series for T (22/94). The “ERC_Conv_” scored the worst for ED (65/176). The “Non-ERC_Conv_” scored the worst for UD visualization (48/91).

### Interobserver variability

For image quality, interobserver variability was *κ* = 0.58 (moderate agreement); for artifacts, interobserver variability was *κ* = 0.34 (fair agreement).

## Discussion

Our results indicate an overwhelming preference for the Non-ERC_DLR_ series by all radiologists (*p* < 0.001). The Non-ERC_DLR_ series best qualitative image score of the four series. Non-ERC_DLR_ was the most frequently selected as the “best series” and the least frequent as “worst series” for most of the anatomical locations evaluations. These results are supportive of our hypothesis that the introduction of DLR may produce improved overall image quality.

The addition of the ERC signal, with either Conventional or DLR, resulted in more overall image artifacts. This was due in part to the pulsation artifacts and/or near field signal artifact seen on the images when the ERC was turned on (Fig. 1) and was interesting that the DLR was not able to remove the artifacts in the presence of ERC. The signal of the ERC was almost 3.8–4.8 times larger on the ERC than on Non-ERC. This benefit of higher signal apparently did not affect the overall image quality as the artifacts obscured the images.

The new MRI PI-RADS 2.1 reporting takes into account image quality and suggests reporting the quality of images. The current bi-parametric MRI relies on T2-weighted and diffusion sequence that is artifact-free and has high quality, and this may be not be achieved in the absence of an ERC [11]. PI-RADS 2.1 advocates using the axial T2 sequence as the key sequence in detecting and staging prostate cancer. Current guidelines, as proposed by the PI-RADS steering committee for an MRI-directed biopsy pathway, also recommend high-quality multiparametric MRI [12], which includes axial T2 as the key sequences. It is therefore important that the image quality of this sequence is optimal. Our study shows that the artifacts from the coil and from rectal motion were significantly reduced by applying the DLR.

DL techniques have been used for reduced noise in brain MRIs [8] and musculoskeletal MRIs [13]. One of the conventional denoising methods is filter-based (i.e., Gaussian filter) noise reduction [8]. However, this approach will not only remove noise but also may result in loss of structural details and create image blurring. The DLR technique used in this work is deep convolutional neural network that operates on raw, complex *k*-space data to reduce image noise, remove truncation artifacts, and improve image sharpness. Instead of directly generating high SNR images, the DLR offers flexibility on noise reduction. This can help avoid aggressive denoising by maintaining a controlled level of noise, making it appear more natural to a human eye.

DL has been used for segmentation of the prostate and urethra [14], for detection of prostate cancer [15], and for radiation treatment planning for prostate cancer [16]. However, to our knowledge, there are no reports of the application of DL or artificial intelligence (AI) techniques to improve image quality and evaluate the anatomy and tumors in patients with prostate cancer. Most of the resources for and research interest in AI associated with prostate MRI have focused on the diagnosis and detection of disease and segmentation of tumors and the prostate. Goldenberg et al. recently published a review article on the role of AI and ML on prostate cancer [17].

There were some limitations to our study. First, we evaluated only a relatively small number of patients. However, our evaluation was performed with three independent reviewers with multiple data points, and the results were overwhelmingly in favor of the “Non-ERC_DLR_” series.

Another limitation was that the tumor, as diagnosed by the radiologists, was not confirmed by slice-by-slice correlation with pathology, although each reader was aware that patients were known to have at least one tumor in the prostate. Based on axial T2W images, the readers selected the “most suspicious area” and compared the same area between series (side to side). By not including the DWI, ADC (apparent diffusion coefficient) map, or other series of the entire MRI study in the data analysis, the readers were not given the full examination for complete interpretation of the MRI images. Our goal, however, was not to assess the accuracy of the radiologists’ visualization of the suspected tumor in a side-to-side comparison. The goal was to evaluate the radiologists’ qualitative analysis of each series in visualization of the suspected tumor in a side-to-side comparison. In the clinical setting, the interpretation of these images requires incorporation of coronal and sagittal T2-weighted series, the DWI/ADC maps, and the pre- and post-Gd images, as required by PI-RADS-2 criteria but not by PI-RADS 2.1 criteria, which requires only T2 axial and one additional T2 orthogonal plane along with diffusion sequences [3].

The other limitation was reader variability for artifacts and overall image quality which were 0.58 and 0.34 (fair to moderate), respectively. This agreement would probably be higher if not for the five options in the scoring of artifacts and the three options in the scoring of image quality. The agreement can be a measure of the reader’s subjectivity: One reader may consider an image acceptable whereas another reader may subjectively consider the same image as excellent; furthermore, one reader may have a higher standard for excellent vs. adequate than another reader. Similarly, one reader may consider a study as having “minimum artifacts” whereas another reader may consider the same study as having “moderate artifacts.” Also we did not allow for more than two series to be selected as “best series.” The radiologists, at times, found three series to be very similar. This limitation, however, did not affect our conclusion that the Non-ERC_DLR_ series was the best for prostate MRI. Also, the Non-ERC_DLR_ images had the ERC in place. While this gave us the opportunity to compare results generated from the same source data thereby removing many confounding factors such as mismatch between images, surface coil positioning differences, and prescan settings variations, we were not able to assess the effect of removing the ERC on overall image quality.

Interestingly, our data seem to indicate that ERC at 3 T may not have the expected added benefit on image quality. The expected improvement in overall signal was observed. The contrast, as scored subjectively by the radiologists, between the anatomical locations did not improve with improvement in the overall signal. We propose that the signal of the ERC did not improve the image quality due to two additional factors: (i) artifacts and (ii) anatomy displacement by the coil. With the ERC turned “off,” the pulsation artifacts from the rectum were diminished (Fig. 1).

## Conclusions

DL Recon provided substantial noise reduction without any noticeable image blurring or loss of resolution in prostate MRI. Given the new PI-RADS 2.1 with its strong recommendation for optimal T2 axial imaging, a prostate MRI study using Non-ERC_DLR_ T2-weighted series generated by removing the signal from the ERC as part of the prostate MRI will have added benefits [3]. As adapted by each individual radiologist, such a series may be used in combination with or independent of the standard T2W series with convention image reconstruction.
